# Genome-Wide CRISPR/Cas9 Screen Identifies New Genes Critical for Defense Against Oxidant Stress in *Toxoplasma gondii*

**DOI:** 10.3389/fmicb.2021.670705

**Published:** 2021-06-07

**Authors:** Yun Chen, Qi Liu, Jun-Xin Xue, Man-Yu Zhang, Xiao-Ling Geng, Quan Wang, Wei Jiang

**Affiliations:** ^1^Shanghai Veterinary Research Institute, Chinese Academy of Agricultural Sciences, Shanghai, China; ^2^Shanghai Customs District P. R. C. China, Shanghai, China

**Keywords:** *Toxoplasma gondii*, antioxidant, oxidative stress, gene knockout, virulence

## Abstract

*Toxoplasma gondii* is one of the most widespread apicomplexans and can cause serious infections in humans and animals. Its antioxidant system plays an important role in defending against oxidant stress imposed by the host. Some genes encoding the antioxidant enzymes of *T. gondii* have been identified; however, critical genes that function in response to reactive oxygen species (ROS) stress are still poorly understood. Here, we performed genome-wide CRISPR/Cas9 loss-of-function screening in the *T. gondii* RH strain to identify potential genes contributing to the ROS stress response. Under hydrogen peroxide treatment, 30 single guide RNAs targeting high-confidence genes were identified, including some known important antioxidant genes such as catalase and peroxiredoxin PRX3. In addition, several previously uncharacterized genes were identified, among which five hypothetical protein-coding genes, namely, *HP1*–*HP5*, were selected for further functional characterization. Targeted deletion of *HP1* in *T. gondii* RH led to significant sensitivity to H_2_O_2_, suggesting that *HP1* is critical for oxidative stress management. Furthermore, loss of *HP1* led to decreased antioxidant capacity, invasion efficiency, and proliferation *in vitro*. *In vivo* results also revealed that the survival time of mice infected with the *HP1*-KO strain was significantly prolonged relative to that of mice infected with the wild-type strain. Altogether, these findings demonstrate that the CRISPR/Cas9 system can be used to identify potential genes critical for oxidative stress management. Furthermore, *HP1* may confer protection against oxidative damage and contributes to *T. gondii* virulence in mice.

## Introduction

*Toxoplasma gondii* is one of the most successful intracellular apicomplexan parasites because it can infect almost any type of nucleated cell in warm-blooded animals (Webster, 2010). Most *T. gondii* infections are asymptomatic or mild symptoms, but infections in immunocompromised patients can cause severe disease manifestations. Infections in pregnancy can cause miscarriage, fetus death, nervous system problems of the newborn. Livestock infection with *T. gondii* can cause considerable economic losses (Blader and Saeij, 2009; Silva and Langoni, 2009). *Toxoplasma* is successful as a widespread pathogen because multiple hosts become infected through ingestion of water or vegetable contaminated with oocysts or undercooked meat containing viable tissue cysts.

*Toxoplasma* infection is a complex process consisting of multiple, independently regulated steps. Within its hosts, *T. gondii* have remarkable ability to avoid immune surveillance and establish infection (Hunter and Sibley, 2012). Many virulence factors of *T. gondii* involved in the regulation of host immune responses and signal transduction have been studied, including the rhoptry proteins (ROP) 5 and 18, which are important for protecting *T. gondii* from clearance by phosphorylating immune-related GTPases in the host (Behnke et al., 2012; Hermanns et al., 2016). TgIST and ROP16 can translocate to the nucleus or cytoplasm and manipulate host gene expression by inhibiting related transcription factors (Ong et al., 2010; Gay et al., 2016). In addition, pathogen infection induced ROS production in macrophages and neutrophils, triggering an oxidative burst (Murray et al., 1985). Upregulation of ROS has serious deleterious effects, including oxidative DNA damage and lipid peroxidation (Packialakshmi and Zhou, 2018); thus, it is toxic to and can inhibit the intracellular proliferation of *T. gondii* (Lajarin et al., 1999; Aline et al., 2002; Xu et al., 2011; MacMicking, 2012). For successful infection in the host cell, *T. gondii* must overcome the reactive oxygen species (ROS) secreted by immune cells (Lajarin et al., 1999; West et al., 2011; Yang et al., 2020). As such, an effective and networked antioxidant system is essential for protecting *T. gondii* from oxygen toxicity and damage, scavenging ROS, and maintaining intracellular redox homeostasis (Kapoor and Banyal, 2009).

To date, the antioxidant enzymes superoxide dismutase (SOD), catalase (CAT), and peroxiredoxins (Prx) have been identified in *T. gondii* (Dincel and Atmaca, 2016). The main function of SOD is to catalyze the conversion of excess superoxide anions (O_2_^–^) into hydrogen peroxide and oxygen (Fukai and Ushio-Fukai, 2011). CAT is a potent H_2_O_2_-detoxifying enzyme that acts downstream of SOD, which can convert H_2_O_2_ into molecules of water and oxygen, thereby helping *T. gondii* evade the macrophage respiratory burst (Ding et al., 2004). In addition, glutathione peroxidases and the Prxs family contribute to the decomposition of H_2_O_2_ (Kaasch and Joiner, 2015). Furthermore, our previous study showed that thioredoxin reductase (TR) is an important antioxidant enzyme and a necessary virulence factor of *T. gondii* with an important role in the resistance to oxidative damage by maintaining thioredoxin (Trx)-reduction state (Kim et al., 2017; Xue et al., 2017). In addition, *T. gondii* uses the thiol-reduction system, including glutathione, Trx, glutaredoxin, and specific reductases, to resist oxidative damage. These enzymes, which are associated with the antioxidant system, play essential roles during parasite survival and infection (Padín-Irizarry et al., 2016). However, it remains unclear whether other unidentified genes are involved in the resistance to host ROS (Boczoń, 2002).

Genome-wide CRISPR/Cas9 screen are powerful tools for identifying essential genes under a specific condition and studying the molecular mechanisms associated with specific phenotypes. Currently, screening with genome-wide CRISPR/Cas9 gene-editing technology has been successfully applied to human cells and microorganisms (Shalem et al., 2014; Han et al., 2018) and has been used to identify previously uncharacterized functional gene in *T. gondii* (Sidik et al., 2016, 2018). For example, the dense granule protein GRA45 is a virulence factor identified using genome-wide screening (Wang et al., 2020). Therefore, genome-wide CRISPR/Cas9 screen can aid in the identification of new antioxidant genes and further reveal the ROS-scavenging mechanisms of *T. gondii*.

In this study, genome-wide CRISPR/Cas9 technology was used to identify new antioxidant-related genes in *T. gondii* under H_2_O_2_-induced oxidative stress, and high-throughput sequencing was used to screen which mutants were lost by the screen. Several genes with significant deletion of single-guide RNAs (sgRNAs) were identified, among which five hypothetical protein (HP)-coding genes were further analyzed. Functional characterization of parasites lacking these five hypothetical genes was performed by assessing their invasion potential, intracellular replication, H_2_O_2_ resistance, ROS levels, and total antioxidant capacity (T-AOC) *in vitro*, as well as their infection potential *in vivo*.

## Materials and Methods

### Cell Culture and *T. gondii* Maintenance

*Toxoplasma gondii* RH parasites and cells were stored in our laboratory. African green monkey kidney (Vero) cells and murine macrophage (Raw264.7) cells were maintained in Dulbecco’s modified Eagle medium (DMEM) supplemented with 10% heat-inactivated fetal bovine serum (FBS), 2 mM glutamine, 100 kU/L streptomycin, and 400 kU/L penicillin (Beijing Solarbio Science & Technology Co., Beijing, China). A *TR* knockout strain (*TR*-KO) was constructed by Xue et al. (2017), and a pseudokinase ROP5 knockout strain (*ROP5*-KO) was constructed as previously described by Xue et al. (2017). All *T. gondii* strains used in this study were passaged on Vero cells maintained at 37°C in a 5% CO_2_ incubator.

### Animals

Eight-weeks-old female Kunming mice were purchased from the Shanghai Jiesijie Experimental Animal Company (Shanghai, China). All animal experimental procedures were performed in strict accordance with the approved guidelines of the Institutional Animal Care and Use Committee of the Shanghai Veterinary Research Institute.

### Plasmids

The CRISPR Knockout Pooled Library (#80636) and plasmids pCas9/chloramphenicol acetyltransferase (#80323) and pCas9/decoy (#80324) were acquired from Addgene (Watertown, MA, United States).

### Experimental Model of H_2_O_2_-Mediated Oxidative Stress

To determine the maximum concentration of H_2_O_2_ that did not affect cell viability, Vero cells were seeded into 96-well plates (2 × 10^5^ cells/mL) and treated with different concentrations of H_2_O_2_ (0, 100, 200, 300, 400, 500, 600, and 800 μM) (Sigma-Aldrich, St. Louis, MO, United States) for 6, 12, 24, and 48 h. Subsequently, cell viability was assessed using a Cell Counting Kit-8 (CCK-8; Vazyme Biotech, Nanjing, China), in accordance with the manufacturer’s instructions. Absorbance of the wells was measured with a microplate reader at 450 nm. Four replicates were set up for each treatment and the percentage of live cells was expressed in comparison to untreated control cells.

Intracellular ROS levels were determined using the Reactive Oxygen Species Detection Assay Kit (Beyotime, Shanghai, China). Briefly, Vero cells were cultured in 6-well plates (1 × 10^6^ cells/well) and cultured for 24 h. The medium was removed and replaced with fresh medium containing different concentrations (100 and 200 μM) of H_2_O_2_. After the treatment for 1, 2, 4, 6, 12, or 24 h, Vero cells were harvested, resuspended in serum-free medium, and incubated with 2′,7′-dichlorofluorescin diacetate (DCFH-DA, 10 μM) for 37°C 30 min. Next, the cells were washed three times with serum-free DMEM to remove excess probe, and the fluorescence intensity was determined at excitation and emission wavelengths of 488 and 525 nm, respectively, in a Synergy 2 fluorescence microplate reader (BioTek Instruments, Winooski, VT, United States).

To establish the timing at which to assess the differences between different strains in their sensitivity to H_2_O_2_, three strains were used. Among them, the *TR*-KO strain was used as a positive control, as it was previously reported to show high sensitivity to H_2_O_2_ treatment (Xue et al., 2017). The RH and *ROP5*-KO strains (*ROP5* is not an antioxidant gene) were used as negative controls. Three extracellular parasite strains (5 × 10^5^ tachyzoites per experimental group) were collected, suspended in serum-free DMEM containing 200 μM H_2_O_2_ for 15, 30, 45, and 60 min at 37°C. After incubation, the H_2_O_2_ was removed by centrifugation and treated tachyzoites were allowed to infect Vero cells in 12-well plates. The survival of each strain was assessed by counting the number of vacuoles containing more than two parasites after 36 h of culture.

The inhibitory effect of H_2_O_2_ on the intracellular parasites was further assessed by counting the number of parasites in the vacuoles. Briefly, Vero cell grown in 12-well plates were infected with 5 × 10^5^ tachyzoites of three strains, and cultured for 12 h. Next, the Vero cells were washed, and complete medium (DMEM + 2% FBS) with 200 μM H_2_O_2_ was added to each well in the treatment groups, for continuous culture. The number of parasites in the vacuoles was counted at 36 h post-invasion. Three independent experiments were conducted in triplicate, and at least 100 cells were counted.

### Genome-Wide CRISPR/Cas9-Mediated Screening

*Toxoplasma gondii* genome-wide CRISPR/Cas9-mediated screening was performed as described by Sidik et al. (2018). Briefly, a Cas9-expressing strain (RH/Cas9) was constructed by co-transfecting the RH strain with the pCas9/chloramphenicol acetyltransferase and pCas9/decoy plasmids. Next, 500 μg of a sgRNA library linearized with the *Ase*I enzyme was transfected into approximately 8 × 10^8^ RH/Cas9 parasites, which were separated into 16 individual 4-mm-gap electroporation cuvettes. Transfected parasites were allowed to infect 16 plates (15 cm^2^) of 80% confluent Vero cells. The medium was removed from each dish 24 h post-infection and replaced with a selective medium containing 3 μM pyrimethamine and 10 μg/mL *DNase*I (Sigma-Aldrich). Following natural egress, parasites were passaged in Vero cells and selected by pyrimethamine for two more generations. All mutants were equally assigned into a test group and a control group. The test group was incubated in DMEM containing 200 μM H_2_O_2_ at 37°C for 30 min, and then the parasites were added to eight 15-cm^2^ dishes of confluent Vero cells. After culturing for 12 h, the medium was replaced with a fresh medium containing 200 μM H_2_O_2_. The parasites were harvested at 48 h after infection (the time at which the mutant parasites nearly egressed from the host cells) and counted. At least 30% of the population was subjected to the same stimulation until the third passage. The experimental procedures and culture conditions of the control group were the same as those of the test group, except that H_2_O_2_ was not used. Parasites from the control group (TOX1) and test group (TOX2) were then harvested and used for genomic DNA extraction (Tiangen Biotech, Beijing, China) and polymerase chain reaction (PCR) amplification of the sgRNA with a barcoding primer (Supplementary Table 1), respectively. The samples were sent to Novogene Technology (Beijing, China) for sequencing on a HiSeq 2500 system (Illumina, San Diego, CA, United States).

The screening data were analyzed as previously described by Sidik et al. (2016). Briefly, sequencing reads were aligned to the sgRNA library sequences (Addgene), and the abundance of each sgRNA was calculated as raw read numbers. The log_2_-fold change between H_2_O_2_ treated and untreated samples was calculated for each sgRNA. Then, the screening score was calculated as the average log_2_-fold change of the top five most abundant sgRNA for each gene in the untreated sample, which ensured the minimal impact of random loss. In addition, the percentage change of sgRNA loss after H_2_O_2_ treatment was further calculated. Thirty sgRNAs with high loss percentages and loss numbers greater than 100 were selected. The final candidate genes were selected from the 30 sgRNA-targeted genes based on a low screening score and low phenotypic score.

### Construction of *CAT*-KO and *HP*-KO Parasites

Knockout strains were constructed as described by Sidik et al. (2016). Briefly, specific sgRNAs targeting *CAT* and *HP*s were used to replace the sgRNA site in the pU6-DHFR plasmid via a ClonExpress MultiS one-step cloning kit (Vazyme Biotech). RH/Cas9 parasites (1 × 10^7^) were transfected with 20 μg of pU6-sgRNA-DHFR containing guides against different *HP* or *CAT*, respectively. The stable mutants were selected with 3 mM pyrimethamine at 24 h post-transfection, and single clones were obtained by limiting dilution. Positive single clones were identified by amplifying the target gene fragments by PCR. All primers used are listed in Supplementary Table 1.

### Viability Assessment of Extracellular Parasites by H_2_O_2_ Sensitivity Assay

To analyze the effect of H_2_O_2_ on the growth of the RH strain and constructed mutants, freshly egressed parasites were collected, passed through 5-μm filters, washed once with serum-free DMEM, and treated with various concentrations of H_2_O_2_ for 30 min at 37°C and 5% CO_2_. Treated and untreated parasites were allowed to invade Vero cells and cultured for 36 h. Parasite viability was determined by counting the number of vacuoles containing more than two parasites under the microscope.

The effect of H_2_O_2_ on the replication of intracellular parasites was also evaluated. Briefly, purified parasites were allowed to infect Vero cells; after 12 h of continuous culture, non-invaded parasites were removed by washing the cells with phosphate-buffered saline (PBS) and placed in a fresh medium containing 200 μM H_2_O_2_. The parasites were continuously cultured in Vero cells for an additional 24 h. The effect of H_2_O_2_ on the replication rate of the parasites was determined by counting the number of parasites per vacuole under the microscope and then averaging the number of parasites per vacuole.

### Detection of ROS, MDA, and T-AOC

To validate the effect of candidate gene deletion on the antioxidant capacity of *T. gondii*, the ROS, malondialdehyde (MDA), and total antioxidant capacity (T-AOC) levels in each strain were evaluated. Purified tachyzoites (1 × 10^7^) were incubated with DCFH-DA (Beyotime) at 37°C for 30 min. These tachyzoites were washed three times with serum-free DMEM, and the total fluorescence intensity was measured with a Synergy 2 fluorescence microplate reader (BioTek Instruments). The level of total ROS in the mutants was calculated as a percentage of that observed for the RH strain. To investigate whether different strains affected the ROS levels secreted by macrophages, *T. gondii*-infected RAW264.7 murine macrophage cells were evaluated. Briefly, RAW264.7 cells were infected with tachyzoites at a ratio of 2:1, and the ROS levels of RAW264.7 cells were detected using DCFH-DA for 3, 6, 12, and 24 h. The percentage compared to ROS levels in control (non-infected) RAW264.6 cells was calculated.

The MDA level in purified tachyzoites was determined using an MDA assay kit (Beyotime), and the absorbance of the lysate was measured at 532 nm. The results are presented as MDA nanomolar per protein milligram. A T-AOC assay kit (Beyotime) was used to determine T-AOC levels in the tachyzoites based on the ferric reducing ability of plasma. All assay procedures and calculation methods were performed in accordance with the manufacturer’s instructions. The experiments were repeated three times.

### Plaque Assays

Vero cells were infected with 500 freshly harvested parasites in a 6-well plate and grown in DMEM containing 2% FBS with or without 200 μM H_2_O_2_. Each strain was assessed in triplicates. The H_2_O_2_-containing medium was removed and replaced with a normal medium 48 h after infection to avoid damaging the cells. Seven days post-transfection, the monolayers were rinsed with PBS, fixed with 4% formaldehyde for 20 min, stained with crystal violet (Beyotime) for 10 min, and washed with water. Plaque formation and plaque numbers were assessed under an Electron microscope (Nikon, Tokyo, Japan).

### Invasion and Proliferation Assays

Carboxyfluorescein diacetate succinimidyl ester (CFDA-SE; Beyotime) was used to evaluate the invasion rates of the constructed knockout strains. The RH and mutant strains were labeled with CFDA-SE at 37°C for 20 min and then washed with PBS three times. Labeled parasites (1 × 10^6^ per well) were added to 6-well culture plates containing 90% confluent Vero cells and incubated at 37°C for 2 h. Non-invasive parasites were rinsed with PBS. After 12 h of culture, the cell monolayer was detached with trypsin-EDTA (0.25%), resuspended in DMEM supplemented with 10% FBS, and analyzed with a Cytomics FC 500 flow cytometer (Beckman Coulter, Brea, CA, United States).

To investigate the replication ability of the parasites, RH and constructed knockout parasites were used to infect Vero cells in a 6-well plate (1 × 10^6^ parasites/well) and incubated under normal growth conditions. Extracellular parasites were rinsed with PBS 12 h post-infection and cultured for more than 24 h. The number of parasites per vacuole was counted under the microscope for 100 vacuoles. Three independent experiments were conducted in triplicates.

### Virulence Testing

Kunming female mice (8 weeks old, *n* = 10) were intraperitoneally injected with purified parasites of the RH or the constructed knockout strains (1 × 10^3^ parasites) or with the same volume of sterile PBS (negative control). The survival of mice upon infection was monitored to evaluate the virulence of the parasites. Cumulative mortality was plotted and analyzed using GraphPad Prism 5 software (GraphPad Software, San Diego, CA, United States).

### Statistical Analysis

Statistical analysis was performed using SPSS version 20 software (SPSS, Inc., Chicago, IL, United States). Significance was determined by one-way analysis of variance, and all results are presented as the means ± standard deviation. *P* < 0.05 was considered to indicate statistically significant differences.

## Results

### Optimization of Oxidative Stress Experimental Model

**FIGURE 1:**
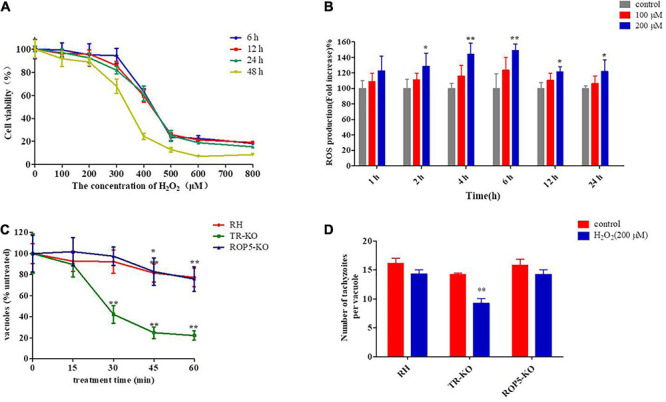
Effect of H_2_O_2_ on Vero cells and different strains of *Toxoplasma gondii*. **(A)** Vero cells were treated with various concentrations of H_2_O_2_ (0, 100, 200, 300, 400, 500, 600, and 800 μM) for 6, 12, 24, and 48 h and then measured using Cell Counting Kit-8. The viability percentage was determined from the absorbance ratio of the treated cells to the control cells (*n* = 4). **(B)** Vero cells were treated with various concentrations of H_2_O_2_ (0,100, and 200 μM) for 1, 2, 4, 6, 12, and 24 h. Intracellular ROS were determined using the Reactive Oxygen Species Detection Assay Kit, and ROS levels are expressed as the fold-change in fluorescence intensity in the test group compared with the untreated group. **(C)** Viability was measured for extracellular parasites (RH, TR-KO, ROP5-KO) treated with 200 μM H_2_O_2_ for 15, 30, 45, and 60 min; next, treated parasites were allowed to infect Vero cells. Vacuoles containing four or more parasites were measured and normalized to the number of untreated parasites incubated at the same time outside of the cell. **(D)** Parasites were treated with or without H_2_O_2_ (200 μM) after 12 h of infection. Parasite replication was quantified for the three strains by counting the number of parasites per vacuole and presented as the mean of parasites per vacuole. The replication assay for the treated group was compared to that for the untreated group. The results are presented as the mean ± standard deviation of three **(B,D)**, four **(A)**, or five **(C)** independent experiments. **P < 0.01, *P < 0.05 significant changes versus control/RH strain.

In previous studies, H_2_O_2_ treatment of extracellular *T. gondii* with antioxidant gene deletion resulted in a remarkable decrease in *T. gondii* viability (Xue et al., 2017). Because *T. gondii* is an obligate intracellular parasite, in addition to treatment with extracellular parasites, we established an oxidative stress model of Vero cells to screen for antioxidant-related genes in *T. gondii*. First, Vero cells were treated with different concentrations of H_2_O_2_, and cell viability was evaluated using the CCK-8 assay. The results demonstrated that H_2_O_2_ inhibited cell viability in a dose- and time-dependent manner (Figure 1A). Cell viability was significantly reduced in the presence of high H_2_O_2_ concentrations (400–800 μM) compared with that in the untreated group (*P* < 0.01 or *P* < 0.05, respectively). A significant decrease in cell viability was also observed after 12 h of treatment with 300 μM H_2_O_2_ (*P* < 0.05). However, the viability of Vero cells treated with 100 or 200 μM H_2_O_2_ was not significantly affected within 24 h and remained higher than 88% after 48 h (91.6 ± 13.34%, 88.81 ± 6.51%). Therefore, these two H_2_O_2_ concentrations (100 and 200 μM) were used in subsequent experiments.

The intracellular ROS level was measured using a Reactive Oxygen Species Detection Assay Kit after 1, 2, 4, 6, 12, or 24 h of H_2_O_2_ treatment. The results revealed significantly increased ROS levels in the 200μM H_2_O_2_ group after 2 h; these levels were maintained at a high level throughout the experimental period (*P* < 0.01 or *P* < 0.05, respectively; Figure 1B). At 100 μM H_2_O_2_, ROS levels were slightly increased compared with that in the control, but the differences were not significant (*P* > 0.05). Accordingly, 200 μM H_2_O_2_, which did not affect cell viability but significantly increased intracellular ROS levels, was used to construct the oxidative stress model for subsequent experiments.

To further determine the optimal incubation time of extracellular parasites with H_2_O_2_ (200 μM), the viability of the three *T. gondii* strains (RH, *ROP5*-KO, and *TR*-KO) was evaluated by counting the number of vacuoles containing more than two parasites. The three strains showed differential sensitivity to H_2_O_2_; the viability of the *TR*-KO strain was significantly inhibited (*P* < 0.01) after incubation with H_2_O_2_ for 30 min, whereas the RH and *ROP5*-KO strains were not susceptible to this condition. The inhibitory effect of H_2_O_2_ on the RH and *ROP5*-KO strains began to increase after 45 min of incubation with H_2_O_2_ (*P* < 0.01 and *P* < 0.05, respectively) (Figure 1C).

The number of parasites per vacuole was calculated to determine the inhibitory effect of H_2_O_2_ on intracellular parasite proliferation. Intracellular parasites were treated with 200 μM H_2_O_2_ for 12 h post-invasion, and the number of parasites in each vacuole was counted 24 h after treatment. Compared with the untreated groups, 200 μM H_2_O_2_ significantly inhibited proliferation of the *TR*-KO strain (*P* < 0.01), whereas it did not affect replication of the RH and *ROP5*-KO strains or Vero cell growth (Figure 1D). Based on these results, the extracellular parasites were treated with H_2_O_2_ for 30 min, and then H_2_O_2_ was added to the medium 12 h after infection and treated continuously until *T. gondii* naturally egressed from the cell, serving as the experimental setting for genomic screening.

### Genome-Wide Screening to Identify Putative Antioxidant Genes in *T. gondii*

**FIGURE 2:**
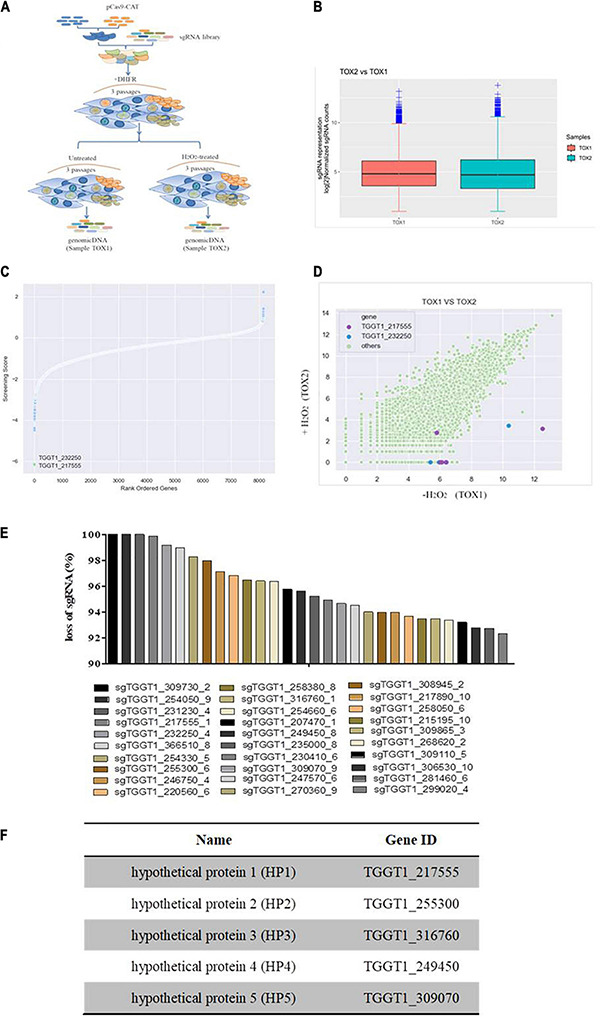
Genome-wide screen identifies mutants hypersensitive to H_2_O_2_. **(A)** Workflow of the genome-wide screen. RH/Cas9 parasites were transfected with a library of sgRNAs. Transfected parasites were grown in Vero cells and selected by pyrimethamine. Subsequently, the mutants were either treated with H_2_O_2_ for three passages or continuously passaged in Vero cells. SgRNA was sequenced and analyzed to select genes related to H_2_O_2_ sensitivity. **(B)** Boxplot showing the distribution of sgRNA frequencies before and after H_2_O_2_ treatment. **(C)** *Toxoplasma gondii* genes were ordered according to their screening scores, which is defined as the mean log_2_-fold-change of the top five most abundant sgRNA for each gene in the control sample. A lower score indicates that the sensitivity of mutants to H_2_O_2_ increases when the gene is deleted. Guides against TGGT1_217555 and CAT were significantly depleted upon H_2_O_2_ treatment. **(D)** Scatter plots representing the relative abundance of sgRNAs in the untreated sample and H_2_O_2_-treated sample; sgRNAs against TGGT1_232250 (blue); sgRNAs against TGGT1_217555 (purple). **(E)** The top 30 candidate genes were selected according to the percentage of lost sgRNA. **(F)** List of selected putative antioxidant genes numbered according to the percentage of lost sgRNA from highest (*HP1*) to lowest (*HP5*).

Cas9-expressing *T. gondii* strains were selected and obtained with 40 mM chloramphenicol. A diverse population of mutants was generated by transfecting a genome-wide sgRNA library containing 10 different sgRNAs against each of the 8,156 genes of this parasite into the Cas9-expressing *T. gondii* strains (RH/Cas9). Based on this mutant pool, loss-of-function screening was performed to select mutants that were susceptible to H_2_O_2_-mediated growth inhibition. This pool was propagated in the presence or absence of 200 μM H_2_O_2_ for three passages, and then, the relative abundance of sgRNAs was measured by Illumina sequencing using the DNA of untreated and treated parasites (Figures 2A,B). The log_2_-fold change of sgRNA between treated and control samples (screening score) was calculated and used to rank all genes (Figure 2C). The guides against *CAT* (TGGT1_232250) and TGGT1_217555 were among the most significantly depleted in the treated samples (Figures 2C,D). This analysis method has some shortcomings in that some sgRNAs (smaller number) are more likely to be lost and the screening scores in this method are lower. In genome-wide CRISPR/Cas9 screening experiments, sgRNA sequences targeting the same gene have different knockdown efficiencies. To ensure maximum loss percentage of at least one sgRNA targeting the same gene, sgRNAs were further ranked according to the loss percentage. A total of 30 sgRNAs with high knockout efficiency, large number of losses, and high loss proportion were selected by combining loss percentage (>92%) and loss number (>100) of sgRNA (Figure 2E). Among all genes, *CAT* (TGGT1_232250), *TR* (TGGT1_309730), and *PRX3* (TGGT1_230410), which are important antioxidant genes, were selected, indicating that the H_2_O_2_-mediated oxidative stress model was successful and effective in selecting relevant genes involved in the antioxidant system of *T. gondii*, further proving the rationality of this selection method. Combined with phenotype score and screening score of the genes, five genes encoding hypothetical proteins, including TGGT1_217555 and the other four genes with low phenotype score and screening score, were selected for subsequent validation tests (Figure 2F). These genes, namely, *HP1*, *HP2*, *HP3*, *HP4*, and *HP5*, lacked functional annotation and respectively encoded previously unstudied *T. gondii* genes (Figure 2F).

### Identification of Genes Related to H_2_O_2_ Sensitivity

**FIGURE 3:**
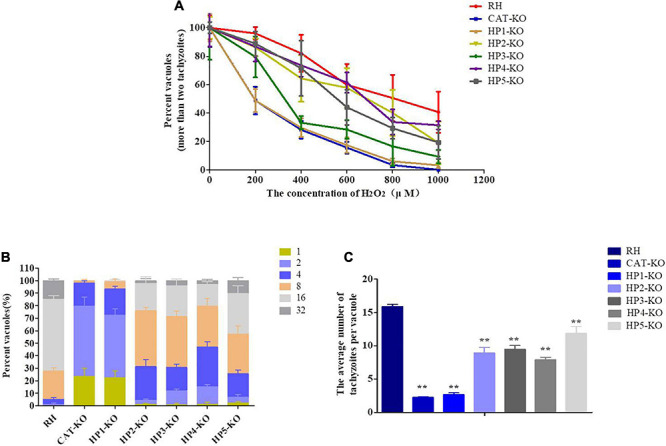
Effect of H_2_O_2_ on knockout parasites and RH strain. **(A)** Effect of H_2_O_2_ on the viability of *T. gondii* parental and knockout strains. Fresh tachyzoites of different strains were incubated for 30 min in the presence of the indicated H_2_O_2_ concentrations. The treated parasites were inoculated on Vero cells. The number of vacuoles was counted under a microscope at 36 h after infection. Data are expressed as a percentage (%) of the number of vacuoles formed by the treated groups compared to the untreated group. **(B,C)** At 12 h post-infection with tachyzoites, the cells were cultured in a medium containing 200 μM H_2_O_2_. The effect of H_2_O_2_ on parasite replication was assessed by calculating the number of tachyzoites in each vacuole after 36 h of infection for a minimum of 100 vacuoles per strain. **(B)** Data are expressed as the percentage of vacuoles containing 1, 2, 4, 8, 16, and 32 parasites. **(C)** Data are expressed as the average number of parasites per vacuole. Data are represented as the mean ± standard deviation (*n* = 4). **P < 0.01, *P < 0.05, significant changes versus controls.

To verify the antioxidant capacity and functionally characterize the candidate genes, *HPs* and *CAT* knockout strains were successfully constructed in the RH/Cas9 strains, and *CAT*-KO strain was used as a positive control. Next, the susceptibilities of wild-type RH and deletion strains to different H_2_O_2_ concentrations were evaluated by measuring the parasite viability, which was determined by counting the number of vacuoles containing more than two parasites after 36 h of culture. The results showed that with an increasing H_2_O_2_ concentration, the inhibition of *T. gondii* viability was enhanced, and the sensitivity of each knockout strain to H_2_O_2_ was increased to varying degrees compared to the RH strain. Particularly, the *CAT*-KO and *HP1*-KO mutants were the most sensitive to H_2_O_2_ stress. The *HP3*-KO strain also showed significantly increased sensitivity to H_2_O_2_ (Figure 3A). In addition, some parasites lacking *CAT* or *HP1* were mostly unable to replicate, even if they were not immediately killed by H_2_O_2_.

Next, the effect of H_2_O_2_ on the replication ability of intracellular parasites was assessed by counting the number of parasites in each vacuole of the RH and mutant strains grown in the presence of 200 μM H_2_O_2_. Overall, the replication ability of each mutant was significantly decreased compared with that of the RH strain (*P* < 0.01). Among these knockout strains, the inhibitory effect of H_2_O_2_ on the replication ability of the *HP1*-KO and *CAT*-KO strains was more significant and higher than 70% for vacuoles containing only one or two parasites (Figures 3B,C). These results revealed that H_2_O_2_ significantly inhibited the intracellular replication of deletion strains and that the inhibitory effect of H_2_O_2_ was most obvious for the *HP1*-KO and *CAT*-KO strains.

### ROS, T-AOC, and MDA Levels in *T. gondii* Mutants and ROS Levels in Mouse Macrophages

**FIGURE 4:**
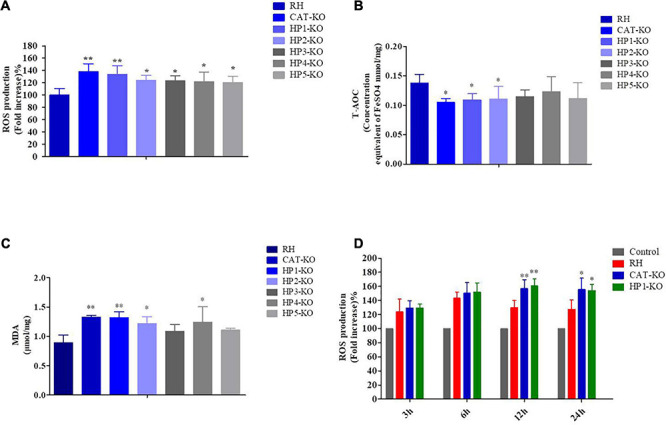
Reactive oxygen species (ROS), total antioxidant capability (T-AOC), and malondialdehyde (MDA) levels in *Toxoplasma gondii* and reactive oxygen species levels in macrophages. **(A)** ROS levels in tachyzoites. Data are expressed as a percentage (%) of the ROS level of mutants compared to the ROS level in RH parasites. **(B)** T-AOC and **(C)** MDA levels in tachyzoites. All samples were measured in triplicates. **(D)** ROS levels in macrophages. Data are expressed as a percentage (%) of ROS levels in mutant-infected macrophages compared to that in control macrophages. All samples were measured in triplicates. **P < 0.01, *P < 0.05 significant changes versus control/RH strain.

As determined by the DCFH-DA assay, the ROS levels in mutants were higher than those in wild-type RH, with the levels in the *CAT*-KO and *HP1*-KO strains significantly higher than those in wild-type RH (*P* < 0.01) (Figure 4A). Similarly, the levels of MDA in all mutants were elevated; MDA levels in the *CAT*-KO, *HP1*-KO, *HP2*-KO, and *HP4*-K*O* strains were significantly higher than those in wild-type RH (*P* < 0.01 or *P* < 0.05) (Figure 4C). Similarly, T-AOC levels in mutants were lower than those in wild-type RH, with significant differences detected between the *CAT*-KO, *HP1*-KO, and *HP2*-KO strains and wild-type RH (*P* < 0.05) (Figure 4B). These results suggest that CAT and HP1 are critical for the antioxidant system of *T. gondii*. Furthermore, the effects of infection by *CAT*-KO, *HP1*-KO, and RH strains on ROS production in mouse macrophages at a 2:1 ratio (tachyzoite: macrophages) were observed. The results showed that ROS levels in macrophages infected with the three strains increased significantly throughout the experimental period. Moreover, ROS levels in macrophages first increased and then tended to decrease with prolonged culture time. Particularly, ROS levels in macrophages infected with *CAT*-KO and *HP1*-KO peaked at 12 h, and there was a significant difference in ROS levels in macrophages infected with *CAT*-KO, *HP1*-KO, and wild-type RH strains at 12 and 24 h (*P* < 0.01 or *P* < 0.05; Figure 4D). The results indicate that *CAT* and *HP1* deletion affects ROS production in *T. gondii*-infected macrophages.

### Phenotypic Analyses of *HP*-KO Strains

**FIGURE 5:**
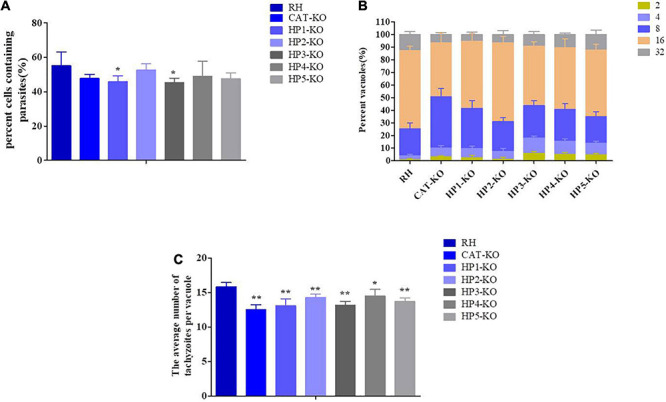
Invasiveness and proliferative potential of hypothetic protein (*HP*)-knockout (KO) *Toxoplasma gondii* strains. **(A)** Invasiveness potential of *HP*-KO strains. **(B,C)** Parasite replication was assessed by calculating the number of tachyzoites in each vacuole after 36 h of infection for a minimum of 100 vacuoles per strain. Data are represented as the mean ± standard deviation (*n* = 4). **P < 0.01, *P < 0.05, significant changes versus controls.

**FIGURE 6:**
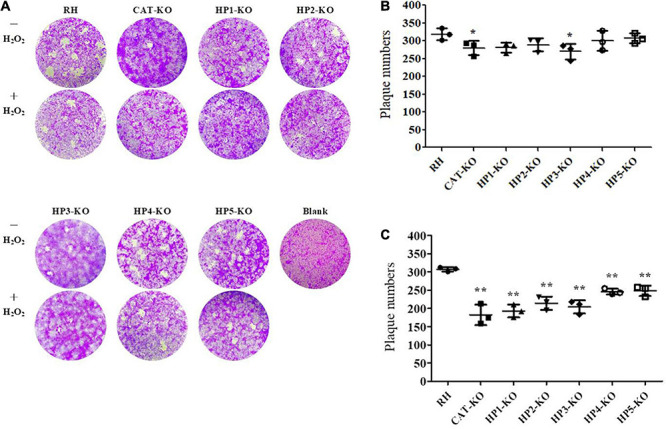
Plaque assays. Vero cells were infected with 500 tachyzoites of each strain and cultured for 7 days. In addition, treated groups were added to H_2_O_2_-containing medium at 12 h after infection, which was replaced with a normal medium at 48 h after infection. After 7 days, plaques were stained with crystal violet and photographed. **(A)** Representative images (magnification: × 100). **(B)** Plaques formed under normal culture conditions. **(C)** Plaques formed under H_2_O_2_ culture conditions. Data for the mutants were compared to the wild-type RH strain.

The invasion capacity of *HP*-KO strains was determined by flow cytometry after 12 h of culture. The mutants showed different degrees of reduced invasiveness and invasion efficiency, which were significantly lower in *HP1*-KO and *HP3*-KO strains than in the wild-type RH (*P* < 0.05; Figure 5A). Moreover, the average number of parasites per vacuole of all mutant strains was significantly lower than that of wild-type RH (*P* < 0.01 or *P* < 0.05) (Figures 5B,C). Next, plaque assays were performed with all mutants on a monolayer of Vero cells to assess parasite viability and competency over several lytic cycles. All *HP*-KO strains had varying degrees of reduced plaque formation, and the plaques formed were smaller than those of the wild-type RH strain, indicating a growth defect (Figure 6A). Comparatively, both *CAT*-KO and *HP3*-KO showed a significant reduction in plaque formation (Figure 6B). The plaque assays were repeated under H_2_O_2_ conditions (medium supplemented with 200 μM H_2_O_2_), in which all mutants formed plaques significantly smaller than those of the wild-type RH strain, and the plaque formation potential in the mutants was significantly reduced (Figures 6A,C). These results revealed that deletion of *HPs* and *CAT* affected parasite proliferation and led to growth defects. Furthermore, the inhibitory effect of H_2_O_2_ on the growth of the *HP*-KO strains was greater than that of the wild-type RH strain. This was particularly noticeable for the *CAT*-KO and *HP1*-KO strains. This finding was consistent with the increase in the ROS levels in mutant strains, further proving that these genes are related to the antioxidant activity of *T. gondii*.

### Effect of *HP* Mutants on *T. gondii* Virulence

**FIGURE 7:**
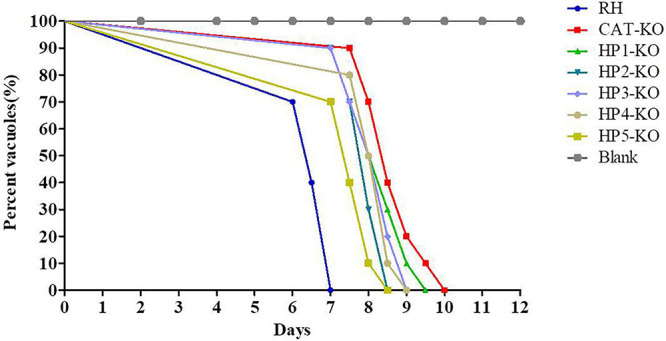
Effect of hypothetic protein (*HP*)-knockout (KO) on *Toxoplasma gondii* virulence. Kunming mice (10 mice/group) were intraperitoneally injected with 1 × 10^3^ parasites of KO strains or RH strain of *T. gondii*, and the survival time of mice was monitored for 12 days.

Previous studies showed that deletion of the antioxidant-related genes of *T. gondii*, including *TR* and *CAT*, attenuated virulence in mice (Ding et al., 2004; Xue et al., 2017). Therefore, we evaluated the influence of *HP* deletion on the virulence of the RH strain. Eight-week-old Kunming mice (10 mice/group) were injected with purified tachyzoites of wild-type RH, *CAT*-KO, or *HP*-KO strains. The survival rates of all mice are shown in Figure 7. Mice inoculated with the RH strain died within 7 days, while mice infected with knockout strains had extended survival times. Remarkably, the survival times of mice infected with the *CAT*-KO and *HP1*-KO strains was significantly longer than those of mice infected with wild-type RH, which died within 7 days of inoculation (*P* < 0.01; Figure 7). These results indicated that *CAT* or *HP1* disruption led to weakened virulence of the parasite.

## Discussion

In the current study, genome-wide loss-of-function screening was performed to identify *Toxoplasma* genes that determine fitness in an H_2_O_2_-induced oxidative stress environment *in vitro*. Evading oxidative damage caused by host ROS is essential for *T. gondii* to establish acute or chronic infection (Arsenijevic et al., 2001; Zhu et al., 2019). As shown in our previous study that TR in *T. gondii* is an essential virulence effector with antioxidant function, which is critical for parasite growth and survival (Xue et al., 2017). Moreover, in-depth studies of *T. gondii* antioxidant-related genes may provide a theoretical basis for developing anti-*T. gondii* drugs because some currently available drugs (such as monensin, mefloquine, and artemisinin) that induce ROS production and target the antioxidant system have been shown to be effective against *T. gondii* and/or *Plasmodium* (Berens et al., 1998; Charvat and Arrizabalaga, 2016; Gunjan et al., 2016; Zhai et al., 2020). Therefore, identification parasite antioxidant genes is important for understanding the mechanisms of *T. gondii* in eliminating ROS, as well as for identifying useful targets for anti-*Toxoplasma* drugs, and this requires further investigations.

Reactive oxygen species consist of O_2_^–^, H_2_O_2_, hydroxyl radical, and ozone (Yang et al., 2020). Being the most stable ROS element, H_2_O_2_ can stimulate the production of endogenous ROS and trigger oxidative damage (Jakovljević et al., 2018). Co-incubation of *T. gondii* mutants with H_2_O_2_ is a strategy for determining whether genes are associated with resistance to ROS (Ding et al., 2004). Herein, H_2_O_2_ was further added to the cell culture medium to enhance the inhibitory effect of H_2_O_2_ on intracellular *T. gondii*. The results revealed no significant effect on the cells or the *T. gondii* RH strain at 200 μM H_2_O_2_, but a significant inhibitory effect on the viability and proliferation of *TR*-KO was noted. This is consistent with the findings of our previous study (Xue et al., 2017). Based on this optimal experimental setting, some genes for which sgRNA numbers were remarkably decreased upon H_2_O_2_ treatment were selected. *CAT*, *TR*, and *PRX3*, associated with ROS resistance (Ding et al., 2004; Xue et al., 2017), were among the identified genes, demonstrating that our H_2_O_2_-mediated oxidative stress screening is a useful approach for identifying ROS-related genes.

Five candidate genes (TGGT1_217555, TGGT1_255300, TGGT1_316760, TGGT1_249450, and TGGT1_309070), showing significantly reduced sgRNA levels upon H_2_O_2_ stimulation, were further investigated. Upon genetic disruption of each HP- and CAT-encoding gene, H_2_O_2_ markedly inhibited the viability and proliferation of the parasites. In addition, increased levels of ROS and MDA and the reduced level of T-AOC in *HP*-KO strains further indicated that these HPs are associated with the antioxidant capacity of *T. gondii*. Moreover, the higher sensitivity of *HP1*-KO to H_2_O_2_ was similar to that of *CAT*-KO, suggesting that HP1 function is critical for resisting ROS. When the activity of antioxidant enzymes, such as CAT, is reduced, the susceptibility of the parasites to oxidative stress increases, and H_2_O_2_-induced ROS can disrupt the redox balance in the parasite, leading to dysfunction of the antioxidant defense and consequent DNA damage, protein damage, or death of the parasite (Shojaee et al., 2014; Lin et al., 2016). Monocytes/macrophages are involved in the defense against intracellular *T. gondii* by upregulating ROS levels (Yamashita et al., 1998; Hunter and Sibley, 2012). We also found that ROS levels in macrophages infected with *HP1*-KO strain were higher than those infected with RH strain, which may increase the inhibitory effect of macrophages on *HP1*-KO strain. However, the mechanisms that lead to increased ROS levels need further research.

Additional experiments demonstrated that the invasion and proliferation abilities of each *HP*-KO strain were decreased compared with those of the wild-type strain and that virulence in mice was attenuated, and the survival time of mice infected with the *HP*-KO and *CAT*-KO strains was longer than that of mice infected with the RH strain. These results suggest that *HPs* are important for the growth of tachyzoites. Plaque assays further indicated that HP disruption caused growth defects of parasites, forming smaller plaques. However, each *HP*-KO strain could be independently grown in normal culture. Further, the virulence of mutants was not completely lost, suggesting that the complex antioxidant mechanisms that involve the interaction between several proteins and molecules, as well as with other antioxidant gene, can confer protection against endogenous oxidative damage. Indeed, SOD2 and a thioredoxin peroxidase found in the apicoplast can eliminate ROS, and PRX1, PRX2, and PRX3 act downstream of SODs to detoxify H_2_O_2_ (Ding et al., 2004; Pino et al., 2007). In *Plasmodium falciparum*, the glutathione and thioredoxin systems are the two main branches of redox homeostasis regulation, with functional complementation between these systems possibly compensating for the lack of some key antioxidant proteins (Chaudhari et al., 2017).

Furthermore, parasites lacking HP1 were more sensitive to externally applied H_2_O_2_ than other *HP*-KO strains, suggesting that HP1 plays an important role in regulating oxidative stress. The precise role of HP1 in *T. gondii* requires further analysis. To date, HP1 is a completely unknown protein with a molecular mass of 11.6 kDa. No signal peptides or known structural domains were found in this protein. *HP1* homologs are only found in Aconoidasida and Coccidia. Only 32% of *HP1* transcripts matched with glucose 6-phosphate dehydrogenase (G6PDH) assembly protein (OpcA) of *Synechococcus elongatus* in the UniProtKB/Swiss-Prot database (details are provided in Supplementary Figure 2). OpcA was reported to be involved in oligomerization and activation of G6PDH. In addition, some thioredoxin regulate G6PDH activity by a change in the OpcA redox status (Özkul and Karakaya, 2015; Mihara et al., 2018). G6PDH is necessary for enhancing the activity of antioxidant enzymes and regulating NADPH provision. Earlier studies found that the G6PDH activity of *Trypanosoma cruzi* was markedly increased under H_2_O_2_ stress (Igoillo-Esteve and Cazzulo, 2006). Additional studies to evaluate the molecular machinery of *HP1* by transcriptome sequencing and protein interaction analysis are needed.

## Conclusion

In conclusion, our study demonstrated that the CRISPR/Cas9 system can be used to identify potential genes involved in oxidative stress response in *T. gondii*. Functional characterization of five hypothetical genes was performed. Our results indicate that HP1 plays an important role in the defense against oxidative damage and can be considered a virulence factor associated with *T. gondii* infection. These results provide broad-based functional information on *T. gondii* genes related to oxidative stress and will facilitate future studies to expand the landscape of anti-parasitic interventions.

## Data Availability Statement

The datasets presented in this study can be found in online repositories. The names of the repository/repositories and accession number(s) can be found below: https://www.ncbi.nlm.nih.gov/, PRJNA707360.

## Ethics Statement

The animal study was reviewed and approved by the Institutional Animal Care and Use Committee of the Shanghai Veterinary Research Institute.

## Author Contributions

YC, J-XX, QW, and WJ designed the research. YC, QL, and QW performed the research. YC, QW, WJ, X-LG, and M-YZ analyzed the data. YC, QL, and WJ wrote the manuscript. All authors contributed to the article and approved the submitted version.

## Conflict of Interest

The authors declare that the research was conducted in the absence of any commercial or financial relationships that could be construed as a potential conflict of interest.

## Supplementary Material

Supplementary Figure 1 PCR validation of CAT- and hypothetical protein (HP)-KO parasites.

Supplementary Figure 2 Bioinformatics analysis of hypothetical protein HP1.

Supplementary Table 1 Primers used in this study.

Supplementary Table 2 Information on the 30 genes selected in the analyses.

Supplementary Data Sheet 1 Raw sgRNA read counts and analysis of sgRNA sequencing data.
