# Supplementary material for: Genome-Wide CRISPR/Cas9 Screen Identifies New Genes Critical for Defense Against Oxidant Stress in Toxoplasma gondii
Source: Front Microbiol. 2021 Jun 7;12:670705. doi: 10.3389/fmicb.2021.670705 (PMC8216390; doi:10.3389/fmicb.2021.670705)
Supplement: Supplementary Figure 1 — PCR validation of CAT- and hypothetical protein (HP)-KO parasites. [file Presentation_1.pdf]

Supplementary Material

2 Supplementary Figures

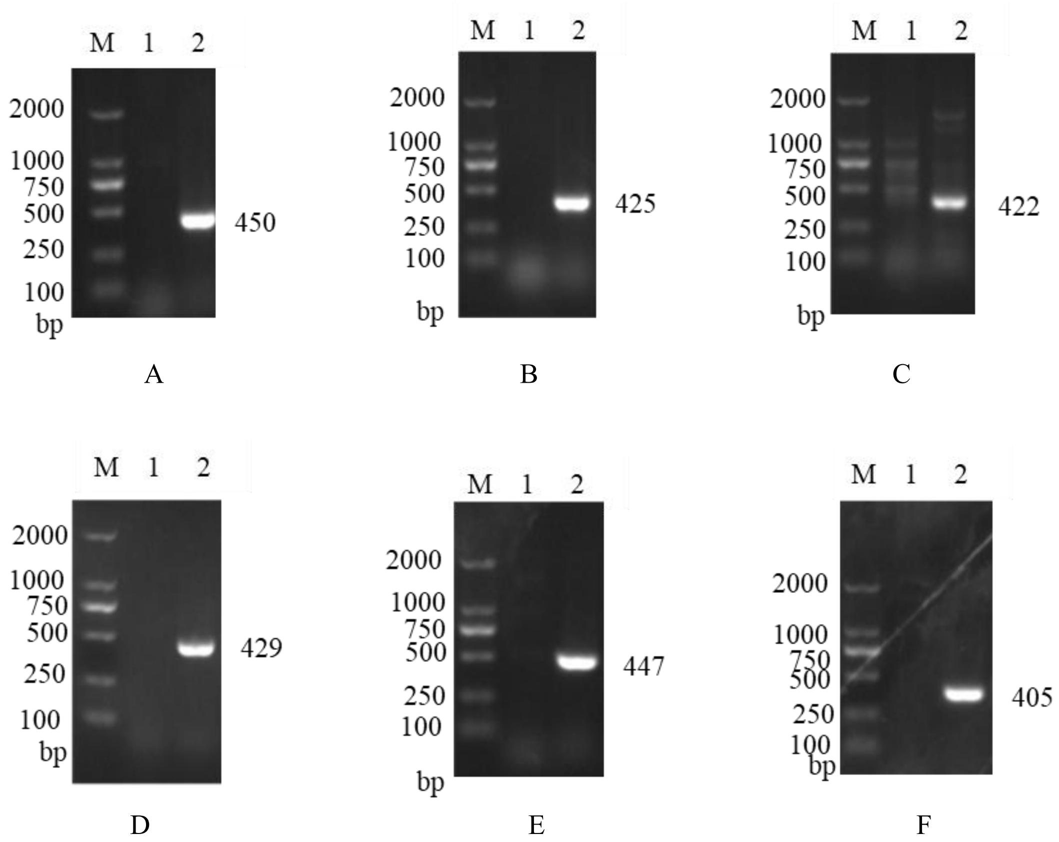

Supplementary Figure 1. PCR validation of CAT- and hypothetical protein (HP)-KO parasites. M:DL2000 DNA Marker; 1:PCR product of target gene in deletion strain; 2: PCR product of target gene in RH strain

A) PCR validation of *CAT* gene deletion. B) PCR validation of *HP1* gene deletion. C) PCR validation of *HP2* gene deletion. D) PCR validation of *HP3* gene deletion. E) PCR validation of *HP4* gene deletion. F) PCR validation of *HP5* gene deletion.
