# Supplementary material for: Genome-Wide CRISPR/Cas9 Screen Identifies New Genes Critical for Defense Against Oxidant Stress in Toxoplasma gondii
Source: Front Microbiol. 2021 Jun 7;12:670705. doi: 10.3389/fmicb.2021.670705 (PMC8216390; doi:10.3389/fmicb.2021.670705)
Supplement: Supplementary Figure 2 — Bioinformatics analysis of hypothetical protein HP1. [file Presentation_2.pdf]

Supplementary Material

A

Transcript ID	Transcript Length	Protein Length	Genomic Length	Isoelectric Point	Molecular Weight	Has SignalP	Has TMHMM
TGGT1_217555	327	108	1346	8.26	11.564 kDa	No	No

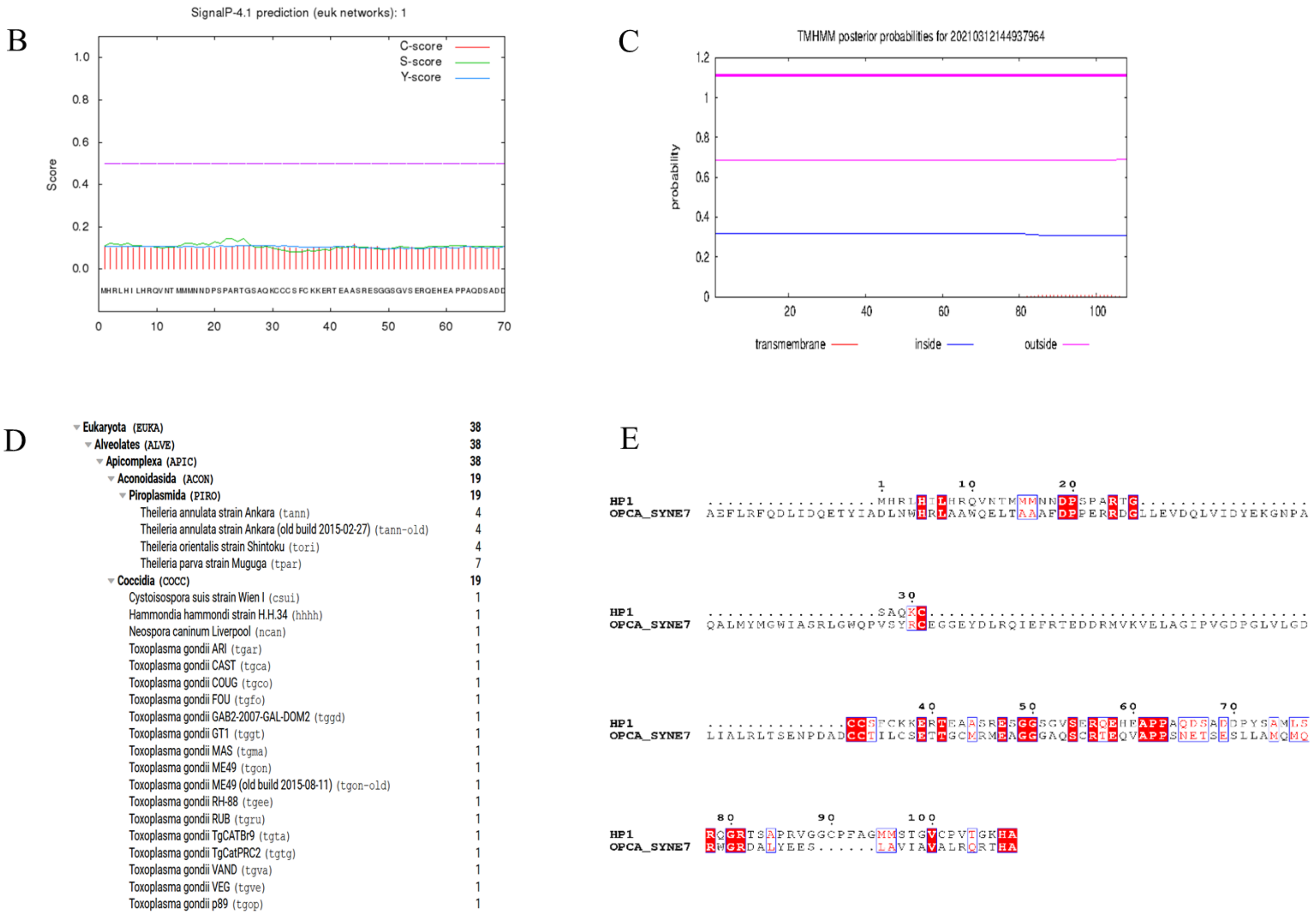

Supplementary Figure 2. Bioinformatics analysis of hypothetical protein HP1; A: Properties and features of HP1; B: Analysis of signal peptide; C: Analysis of transmembrane region; D: Homologous analysis of HP1; E: Alignment of amino acid sequence of TgHP1 with OPCA of *Synechococcus elongatus*
