# Supplementary material for: Genome-Wide CRISPR/Cas9 Screen Identifies New Genes Critical for Defense Against Oxidant Stress in Toxoplasma gondii
Source: Front Microbiol. 2021 Jun 7;12:670705. doi: 10.3389/fmicb.2021.670705 (PMC8216390; doi:10.3389/fmicb.2021.670705)
Supplement: Supplementary Table 1 — Primers used in this study. [file Table_1.DOCX]

Supplementary Material

# Supplementary Table

**Supplementary Table S1.** Primers used in this study

| name | Sequence (5' to 3') | use |
| --- | --- | --- |
| Sequence F | CTCTGACAAGAATCATATTTCCATC | amplifying sgRNA library |
| Sequence R | ACCGACTCGGTGCCACTTTT | amplifying sgRNA library |
| CAT 1F | gggaaactctcgcttggcaaGTTTTAGAGCTAGAA | sgRNA to disrupt TGGT1_232250 |
| CAT 1R | GCAGGGCTCTAGAACTAGTGGATCG | cloning pU6-DHFR |
| CAT 2F | CGATCCACTAGTTCTAGAGCCCTGC | cloning pU6-DHFR |
| CAT 2R | ttgccaagcgagagtttcccAACTTGACATCCCCATTTAC | sgRNA to disrupt TGGT1_232250 |
| HP1 1F | gcagcaagtcgcgaatccggGTTTTAGAGCTAGAA | sgRNA to disrupt TGGT1_217555 |
| HP1 2R | ccggattcgcgacttgctgcAACTTGACATCCCCATTTAC | sgRNA to disrupt TGGT1_217555 |
| HP2 1F | gtctccgtgcaaagaagcgtGTTTTAGAGCTAGAA | sgRNA to disrupt TGGT1_255300 |
| HP2 2R | acgcttctttgcacggagacAACTTGACATCCCCATTTAC | sgRNA to disrupt TGGT1_255300 |
| HP3 1F | gagtagaacatccttctcggGTTTTAGAGCTAGAA | sgRNA to disrupt TGGT1_316760 |
| HP3 2R | ccgagaaggatgttctactcAACTTGACATCCCCATTTAC | sgRNA to disrupt TGGT1_316760 |
| HP4 1F | gacattcgctgcattcggtgGTTTTAGAGCTAGAA | sgRNA to disrupt TGGT1_249450 |
| HP4 2R | caccgaatgcagcgaatgtcAACTTGACATCCCCATTTAC | sgRNA to disrupt TGGT1_249450 |
| HP5 1F | gaaaagctacatggccgggaGTTTTAGAGCTAGAA | sgRNA to disrupt TGGT1_309070 |
| HP5 2R | tcccggccatgtagcttttcAACTTGACATCCCCATTTAC | sgRNA to disrupt TGGT1_309070 |
| pCAT F | ATTGTGTCTCTTTCGCCTGATGCTT | amplifying the CAT locus |
| pCAT R | AAACCCGTTAATCGGCGTGGTTTTC | amplifying the CAT locus |
| pHP1 F | ATATTGGTAGGAGTGTGTTGAGGGG | amplifying the HP1 locus |
| pHP1 R | GTAACACCTAGTAGCATTTGCTGGC | amplifying the HP1 locus |
| pHP2 F | TGGGAGAGACGGAGAGAGCAGAGGA | amplifying the HP2 locus |
| pHP2 R | CTGGGGCGTCCTCTCCGCTATCTCA | amplifying the HP2 locus |
| pHP3 F | ATGCTCGGATCTCGGCTTCGCCACG | amplifying the HP3 locus |
| pHP3 R | ATTGGCACTTCCGGTCCCCGTGTGG | amplifying the HP3 locus |
| pHP4 F | GAACGCAGTTGAGCGTCTGCTCTTG | amplifying the HP4 locus |
| pHP4 R | CTCTCGTTCTGTCGCCTCTCTTCCT | amplifying the HP4 locus |
| pHP5 F | AGACCGCTGGGAGGGAACAGAGTGG | amplifying the HP5 locus |
| pHP5 R | TCGACTCAGTGTGCAACGACGCAGG | amplifying the HP5 locus |
