# Supplementary material for: Genome-Wide CRISPR/Cas9 Screen Identifies New Genes Critical for Defense Against Oxidant Stress in Toxoplasma gondii
Source: Front Microbiol. 2021 Jun 7;12:670705. doi: 10.3389/fmicb.2021.670705 (PMC8216390; doi:10.3389/fmicb.2021.670705)
Supplement: Supplementary Table 2 — Information on the 30 genes selected in the analyses. [file Table_2.docx]

| sgRNA ID | Gene ID | Annotation | sgRNA number | | Loss number | Loss percentage | H_2_O_2_  Screening Score | Phenotype Score |
| --- | --- | --- | --- | --- | --- | --- | --- | --- |
|  |  |  | TOX1 | TOX2 |  |  |  |  |
| sgTGGT1_309730_2 | TGGT1_309730 | thioredoxin reductase | 137 | 0 | 137 | 100 | -3.97 | -1.98 |
| sgTGGT1_254050_9 | TGGT1_254050 | optic atrophy 3 protein (opa3) protein | 110 | 0 | 110 | 100 | -3.82 | 0.39 |
| sgTGGT1_231230_4 | TGGT1_231230 | hypothetical protein | 105 | 0 | 105 | 100 | -1.58 | -0.13 |
| sgTGGT1_217555_1 | TGGT1_217555 | hypothetical protein 1 | 5976 | 9 | 5967 | 99.85 | -6.17 | 1.6 |
| sgTGGT1_232250_4 | TGGT1_232250 | catalase | 1339 | 11 | 1328 | 99.18 | -6.15 | 2.08 |
| sgTGGT1_366510_8 | TGGT1_366510 | hypothetical protein | 2461 | 26 | 2435 | 98.94 | -0.62 | 0.78 |
| sgTGGT1_254330_5 | TGGT1_254330 | lipase | 116 | 2 | 114 | 98.28 | -1.36 | 1.44 |
| sgTGGT1_255300_6 | TGGT1_255300 | hypothetical protein 2 | 103 | 2 | 101 | 98.06 | -3.83 | -2.99 |
| sgTGGT1_246750_4 | TGGT1_246750 | hypothetical protein | 104 | 3 | 101 | 97.12 | -1.23 | 0.06 |
| sgTGGT1_220560_6 | TGGT1_220560 | hypothetical protein | 116 | 4 | 112 | 96.55 | -1.13 | -0.85 |
| sgTGGT1_258380_8 | TGGT1_258380 | elongation factor p (ef-p) kow family domain-containing protein | 226 | 8 | 218 | 96.46 | -2.61 | 0.07 |
| sgTGGT1_316760_1 | TGGT1_316760 | hypothetical protein 3 | 139 | 5 | 134 | 96.40 | -1.60 | -5.16 |
| sgTGGT1_254660_6 | TGGT1_254660 | ankyrin repeat-containing protein | 165 | 6 | 159 | 96.36 | -0.67 | -0.07 |
| sgTGGT1_207470_1 | TGGT1_207470 | hypothetical protein | 236 | 10 | 226 | 95.76 | -1.09 | 0.03 |
| sgTGGT1_249450_8 | TGGT1_249450 | hypothetical protein 4 | 114 | 5 | 109 | 95.61 | -2.24 | -1.71 |
| sgTGGT1_235000_8 | TGGT1_235000 | phosphorylase family protein | 105 | 5 | 100 | 95.24 | -1.08 | 0.01 |
| sgTGGT1_230410_6 | TGGT1_230410 | peroxiredoxin PRX3 | 158 | 8 | 150 | 94.94 | -3.01 | 0.4 |
| sgTGGT1_309070_9 | TGGT1_309070 | hypothetical protein 5 | 375 | 20 | 355 | 94.67 | -2.06 | -1.91 |
| sgTGGT1_247570_6 | TGGT1_247570 | hypothetical protein | 110 | 6 | 104 | 94.55 | -0.81 | 0.63 |
| sgTGGT1_270360_9 | TGGT1_270360 | hypothetical protein | 217 | 13 | 204 | 94.01 | -1.42 | 0.32 |
| sgTGGT1_308945_2 | TGGT1_308945 | hypothetical protein | 117 | 7 | 110 | 94.01 | -1.44 | -0.79 |
| sgTGGT1_217890_10 | TGGT1_217890 | putative alkyl hydroperoxide reductase | 116 | 7 | 109 | 93.97 | -3.08 | 0.23 |
| sgTGGT1_258050_6 | TGGT1_258050 | actin like protein ALP2a | 142 | 9 | 133 | 93.66 | -1.13 | -4.44 |
| sgTGGT1_215195_10 | TGGT1_215195 | tetratricopeptide repeat-containing protein | 123 | 8 | 115 | 93.50 | -1.23 | 1.43 |
| sgTGGT1_309865_3 | TGGT1_309865 | hypothetical protein | 123 | 8 | 115 | 93.50 | -0.84 | 2.85 |
| sgTGGT1_268620_2 | TGGT1_268620 | putative blood stage antigen 41-3 precursor | 136 | 9 | 127 | 93.38 | -1.02 | 1.23 |
| sgTGGT1_309110_5 | TGGT1_309110 | tRNA methyl transferase | 118 | 8 | 110 | 93.24 | -2.18 | -2.38 |
| sgTGGT1_306530_10 | TGGT1_306530 | hypothetical protein | 208 | 15 | 193 | 92.79 | -1.56 | -1.32 |
| sgTGGT1_281460_6 | TGGT1_281460 | hypothetical protein | 110 | 8 | 102 | 92.73 | -0.48 | 0.71 |
| sgTGGT1_299020_4 | TGGT1_299020 | AP2 domain transcription factor AP2III-4 | 130 | 10 | 120 | 92.31 | -0.43 | -0.59 |
